# Using Association Mapping in Teosinte to Investigate the Function of Maize Selection-Candidate Genes

**DOI:** 10.1371/journal.pone.0008227

**Published:** 2009-12-09

**Authors:** Allison L. Weber, Qiong Zhao, Michael D. McMullen, John F. Doebley

**Affiliations:** 1 Laboratory of Genetics, University of Wisconsin-Madison, Madison, Wisconsin, United States of America; 2 Plant Genetics Research Unit, United States Department of Agriculture, Agricultural Research Service and Division of Plant Sciences, University of Missouri, Columbia, Missouri, United States of America; University of Umeå, Sweden

## Abstract

**Background:**

Large-scale screens of the maize genome identified 48 genes that show the putative signature of artificial selection during maize domestication or improvement. These selection-candidate genes may act as quantitative trait loci (QTL) that control the phenotypic differences between maize and its progenitor, teosinte. The selection-candidate genes appear to be located closer in the genome to domestication QTL than expected by chance.

**Methods and Findings:**

As a step toward defining the traits controlled by these genes, we performed phenotype-genotype association mapping in teosinte for 32 of the 48 plus three other selection-candidate genes. Our analyses assayed 32 phenotypic traits, many of which were altered during maize domestication or improvement. We observed several significant associations between SNPs in the selection-candidate genes and trait variation in teosinte. These included two associations that surpassed the Bonferroni correction and five instances where a gene significantly associated with the same trait in both of our association mapping panels. Despite these significant associations, when compared as a group the selection-candidate genes performed no better than randomly chosen genes.

**Conclusions:**

Our results suggest association analyses can be helpful for identifying traits under the control of selection-candidate genes. Indeed, we present evidence for new functions for several selection-candidate genes. However, with the current set of selection-candidate genes and our association mapping strategy, we found very few significant associations overall and no more than we would have found with randomly chosen genes. We discuss possible reasons that a large number of significant genotype-phenotype associations were not discovered.

## Introduction

Past natural or artificial selection leaves its signature on the genome by altering the levels and pattern of nucleotide diversity in a population or species. Advances in high-throughput genotyping and sequencing have enabled large-scale and genomic-wide scans for the signature of selection which identify sets of candidate genes that were putative targets of selection during the history of a population or species [1], [2]. This approach represents a promising way of identifying genes controlling traits important for adaptation in natural species or agronomic traits in crops. An attractive feature of selection scans is that they may identify genes controlling traits that investigators may not have considered important *a priori* [3].

After a “selection-candidate” gene has been identified, a much greater effort will be required to identify the specific phenotype that the gene controls and how variation in the selection-candidate affects the phenotype. If the selection-candidate is of unknown function, one will need to start from scratch with QTL mapping, association mapping, gene expression assays, and gene-knock-outs to address this question. If the selection-candidate belongs to a class of genes of known function, the above types of experiments would still be necessary, although the knowledge of the general function of the class of genes could be used to guide the experiments. Remarkably, even if the selection-candidate is a well-characterized gene, the genotype-phenotype link can remain obscure. For example, *ramosa1* (*ra1*) of maize shows strong evidence that it was the target of selection during maize domestication, and this gene has been characterized in considerable detail [4]. *ra1* encodes a Cys_2_-His_2_ zinc-finger transcription factor belonging to the plant-specific EPF subclass and it functions to impose determinacy on second-order meristems. The difficulty is that the determinacy of second-order meristems was not altered during maize domestication, and so exactly what trait was under selection and how *ra1* affects that trait is not yet known.

Recently, two large-scale selection screens in maize assayed a total of 1869 genes for evidence that they were targets of selection during maize domestication or improvement [5], [6]. Wright *et al.* (2005) identified 30 selected genes using a coalescent likelihood framework to assess loss of single nucleotide (SNP) diversity in maize as compared to teosinte in 774 genes [5]. SNP diversity was assayed by sequencing 100 to 900 bp of genomic sequence in up to 14 maize and 16 teosinte inbred lines. The coalescent likelihood framework assumed two classes of genes, genes affected by the domestication bottleneck alone and a second group of genes that went through a more severe bottleneck representing the effects of both the domestication bottleneck and selection. The analysis calculated the posterior probability of a gene belonging to the selected class.

The second study [6], initially sequenced 1095 genes in up to 14 maize inbreds and then conducted additional sequence analysis on 35 genes that had no genetic diversity as assayed with 200 bp or more of genomic sequence in the 14 maize inbreds. The additional analysis involved obtaining sequence data for the 35 genes from up to 16 teosinte inbreds and 16 maize landraces, and using this data to conduct both the Hudson-Kreitman-Aguadé (HKA) test and coalescent simulations of domestication (CS) tests [7], [8]. These tests identified eight of the 35 genes as potential targets of selection.

Together, these studies identified approximately 48 selection-candidate genes belonging to a broad range of functional classes as well as some genes of unknown function. These 48 genes may contribute to differences in morphology between maize and its progenitor since their genomic locations appear to be closer to domestication QTL for plant and inflorescence morphology than expected by chance [5]. However, for none of these 48 genes is there a clear link between gene function and a specific trait that was under selection during domestication. Thus, to elucidate and confirm the role of these genes in maize domestication, experiments such as QTL mapping, association mapping, gene expression assays, and gene-knock-outs will be required.

As a step toward identifying the traits that these 48 selection-candidates potentially affect, we conducted genotype-phenotype association mapping [9], [10]. We performed the association mapping in the maize progenitor, teosinte, since maize is expected to have little or no variation in these genes. We assayed 82 SNPs located in 32 of the 48 published selection-candidate genes plus three additional selection-candidates identified by the Maize Functional Diversity Project (www.panzea.org) for a total of 35 genes. We used two teosinte association mapping panels in which 32 traits were scored. These traits include many that define the fundamental morphological differences between maize and teosinte. We observed significant associations involving SNPs in seven genes and six different traits. Although we were able to detect seven significant associations, overall the selection-candidate genes were no more likely to associate with trait variation in teosinte than genes chosen at random from the genome. We discuss several reasons why this study did not uncover more significant associations between genetic variation in selection-candidates and trait variation in teosinte. Despite the fact that the success of this study was limited, we still feel that association mapping is a useful approach in identifying the function of selection-candidate genes.

## Results

We used a mixed linear model to test for association between SNP variation in 35 selection-candidate genes and trait variation in teosinte [11], [12]. Our teosinte sample includes two previously described panels: Panel A consists of 584 plants sampled from 74 local populations [13], and Panel B consists of 817 plants from 34 local populations [14]. Twenty-four of the 35 selection-candidate genes were those identified by Wright *et al.* (2005) [5], eight were identified by Yamasaki *et al*. (2005) [6], and three were identified using sequence data from the Maize Functional Diversity Project (www.panzea.org). Traits assayed included those measuring flowering time, plant architecture, inflorescence architecture, vegetative morphology and kernel composition (Table 1; Table S1, Supplementary section). Most of these traits measure aspects of morphology that were altered during maize domestication and/or improvement. The two association panels were analyzed separately. For Panel A, 56 SNPs from 26 selection-candidate genes were tested for association with 17 traits, and for Panel B, 75 SNPs from 35 selection-candidate genes were tested for association with 31 traits (Table S2, Supplementary section). Overall, 82 SNPs in 35 selection-candidate genes and 32 traits were assayed.

**Table 1:** List of traits with repeated associations or associations that survive correction for multiple testing.

Trait	Description^a^	Units	Study
DSCT (derived starch content)	Derived percent dry matter of starch: percent dry matter not accounted for by oil content, protein content and pooled ash and fiber content estimates	percent	Panels A & B
FCWT (fruitcase weight)	Average fruitcase weight based on 50 mature fruitcases	g	Panels A & B
NMFC (number of fruitcases)	Number of female and hermaphroditic cupules in the basal-most ear on the lateral branch	count	Panels A & B
OLCT (oil content)	Percent oil per gram of seed	percent	Panels A & B
PESP (percent pedicellate spikelets)	Percent of fruitcases that have a pistillate spikelet and a staminate spikelet; this trait was measured on bulk seed harvested from the mature plant	percent	Panel B
POLL (days to pollen)	Days from planting to first visible anthers on a single plant	days	Panels A & B

^a^For further detail on the traits and their measurement, see Weber *et al.* 2007 and 2008.

In both panels, all SNPs were tested against all traits giving a total of 3277 SNP-trait pairs tested. Among these SNP-trait pairs, 152 (4.69%) detectable associations (*P*<0.05) were observed, and 35 (1.07%) of these associations had a *P*-value of less than 0.01 (Table S3, Supplementary section). These values are similar to what would be expected under the null hypothesis of independence between the SNP and the trait, *i.e.*, approximately 5% of the associations have a *P*-value of less than or equal to 0.05 and approximately 1% have a *P*-value of less than or equal to 0.01. Only two associations survive a Bonferroni correction for multiple testing (Table 2): an association between a marker in AY104948, a gene homologous by sequence to *ARF2* in Arabidopsis and days to pollen (POLL) [15], and an association between a marker in AY107903, a gene that encodes a ubiquitin C-terminal hydrolase family protein and percent pedicellate spikelets in the ear (PESP), which is a domestication trait.

**Table 2:** Associations that were repeated or survived multiple testing.

Gene	Marker	Trait	Panel	*P*
AY104530	PZA02949.26	POLL	A	0.0450
	PZA02949.22	POLL	B	0.0432
AY104948	PZA02856.1	POLL	B	0.0002
AY106616	PZA03774.9	DSCT	A	0.0453
	PZA03774.9	DSCT	B	0.0381
	PZA03774.10	OLCT	A	0.0109
	PZA03774.10	OLCT	B	0.0282
AY107195	PZA03775.3	POLL	A	0.0246
	PZA03775.3	POLL	B	0.0132
	PZA03775.4	POLL	B	0.0150
	PZA03775.9	NMFC	A	0.0452
	PZA03775.4	NMFC	B	0.0239
AY107903	PZA00407.9	PESP	B	7.09×10^−5^
AY107952	PZA03781.3	NMFC	A	0.0422
	PZA03781.5	NMFC	B	0.0494
AY110082	PZA00170.3	FCWT	A	0.0398
	PZA00170.1	FCWT	B	0.0317

In addition to the associations with two genes that survive the Bonferroni correction, five other genes are notable because they associate (*P*<0.05) with the same trait in both teosinte panels (Table 2). SNPs in AY104530, a serine/threonine kinase, and AY107195, a gene homologous by sequence to *ARF1* in Arabidopsis [16], associate with days to pollen (POLL) in both Panel A and Panel B. SNPs in AY107195 were also found to associate in both panels with the number of fruitcases in the ear (NMFC), which is a domestication trait. AY107952 also associates with NMFC in both panels and has homology to a gene that is expressed early in fruit development of kiwi but has no known molecular function [17]. SNPs in AY110082, a putative heat shock protein, associate with fruitcase weight, another domestication trait. Lastly, AY106616, an ankyrin repeat-like protein, associates with both derived kernel starch content (DSCT) and oil content (OLCT) in both panels.

**Figure 1:**
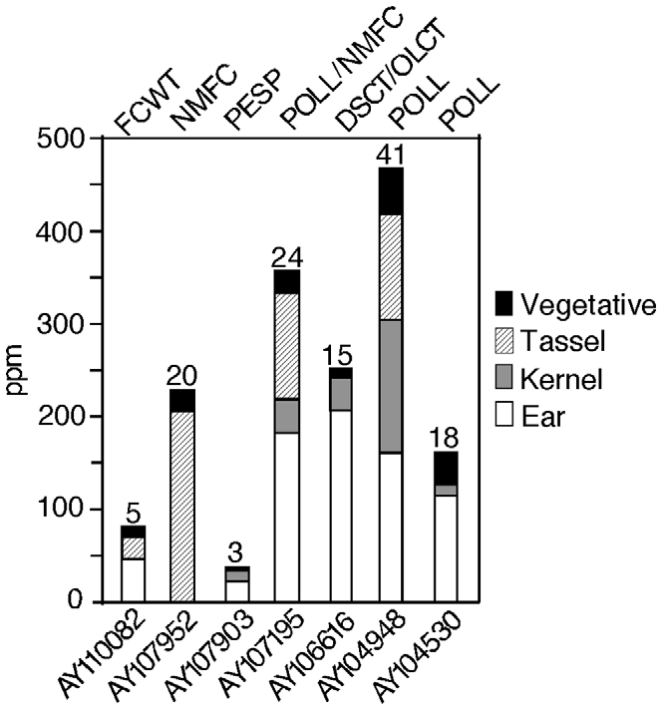
Expression levels of genes that associate with trait variation in teosinte in different tissue types. Expressions levels of the four tissues (ear, kernel, tassel and vegetative) were estimated using an e-northern analysis. Shading indicates tissue type. Expression level is given in parts per million. At the top of each column the number of blast hits is shown. The phenotype that each gene associated with is listed at the top of the graph. For six of the seven genes, the expression pattern was consistent with the trait(s) found to associate with that gene.

To determine the general expression pattern of the seven genes with significant associations, we conducted an electronic northern (e-northern) analysis (Figure 1). For six of the seven genes, the expression pattern was consistent with the trait(s) found to associate with that gene. For example, AY107903, which associates with the number of pedicellate spikelets in the ear (PESP), is predominantly expressed in the ear. AY110082, which associates with fruitcase weight, is also predominantly expressed in the ear, which is homolgous to a group of teosinte fruitcases. AY107195, which associates with number of fruitcases in the teosinte ear (NMFC), is expressed in the maize ear. AY107195 also associates with flowering time (POLL) and it is expressed in vegetative tissue where the initial signaling for flowering occurs [18], [19]. Similarly, the two other genes that associate with POLL, AY104948 and AY104530, are also expressed in vegetative tissue. Lastly, AY106616 a gene that associates with two kernel composition traits, derived starch content (DSCT) and oil content (OLCT), is expressed in the kernel. Only AY107952, which associated with the ear trait NMFC, did not have an expression pattern consistent with this trait since the e-northern data did not show expression of this gene in the ear as expected.

**Figure 2:**
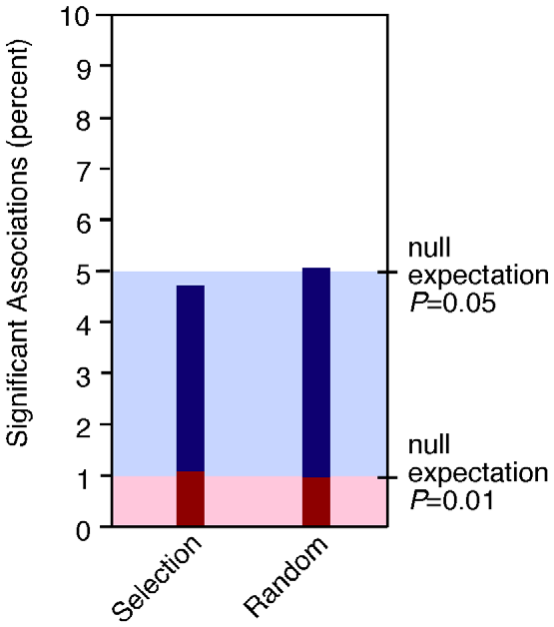
Graph of the percent of association tests observed to be significant. The columns represent the percent of significant association tests (*P*<0.05) observed for each specific group of genes (selection-candidate or random). The light blue shading indicates the expected percent of associations (5.0%) with a *P*-value of less than 0.05 under the null hypothesis of independence between the SNP and the trait. The red areas of the columns designate the subset of significant associations with a *P*-value of less than 0.01 for each specific group of genes. The red shading indicates the expected percent of associations (1.0%) with a *P*-value of less than 0.01 under the null hypothesis of independence between the SNP and the trait. Both the selection-candidates and the random genes had no more significant associations with trait variation in teosinte than would be expected under the null hypothesis.

Overall, the selection-candidate genes showed few associations with traits that measure the morphological differences between maize and teosinte despite the fact that these genes appear to map near QTL for these traits in the genome [5]. For comparison, we conducted permutation tests to determine if selection-candidate genes are more likely to associate with trait variation in teosinte than a comparable sample of random genes. We selected 56 SNPs from the 706 random SNPs previously assayed in Panel A [13]. Similarly, we selected 75 SNPs from the 498 random SNPs previously assayed in Panel B [14]. These random SNPs were chosen based on major allele frequency and the number of SNPs assayed per gene in order to select a group of SNPs similar to those assayed for the selection-candidate genes. A comparison of sequence diversity between the selection-candidates and the random genes as measured by nucleotide polymorphism, (θ) [20] and nucleotide diversity (π) [21] indicated that the two samples of genes were not significantly different as evaluated by the Mann-Whitney (MW) test (θ, *P* = 0.4317; π, *P* = 0.8135). If one defines a significant association using *P*<0.05, then selection-candidate genes associate with traits in 4.69% of tests, while random genes associate with traits in 5.04% of tests (Figure 2), a difference that is not statistically significant by a permutation test (*P* = 0.0850). If one defines a significant association using *P*<0.01, then selection-candidate genes associate with traits in 1.07% of tests, while random genes associate in 1.01% of tests (Figure 2), a difference that is also not statistically significant by a permutation test (*P* = 0.5469). Both of these tests indicate that our selection-candidates are no more likely to associate with teosinte trait variation than a sample of genes chosen at random from the genome.

## Discussion

Our association mapping study involving 35 selection-candidate genes, 32 traits and two teosinte association mapping panels found seven significant associations between selection-candidate genes and trait variation in teosinte. These associations should be regarded with caution and additional molecular work will be necessary to validate them. Interesting associations were detected between the maize homologs of two Arabidopsis genes, *ARF1* and *ARF2*, and days to pollen (POLL). Mutant analysis in Arabidopsis indicates that both these genes affect flowering time [22]. Curiously, flowering time was not a trait hypothesized to have been under selection during maize domestication, and thus these putative associations may be independent of the selective history of these genes. Associations were also detected between AY106616, an ankyrin-repeat like protein, and derived starch content (DSCT) and oil content (OLCT). Previous studies in Arabidopsis and tobacco indicate that ankyrin-repeat like proteins are involved in carbohydrate metabolism and allocation [23], [24]. These significant associations, as well as those involving less well-annotated genes, provide information that can now be used as a starting point for further experimentation that will validate the function of these genes.

These significant associations are not the first to be detected between markers in genes that underwent selection during domestication and trait variation in teosinte. Our previous studies [13], [14] have identified several selected genes that affect trait variation in teosinte. In each of these cases, the evidence for selection on the gene was very strong. *tb1,* a domestication gene of large effect [25]–[27], was found to associate with variation in both plant and inflorescence architecture in teosinte. Three other selected genes that were found to associate with teosinte trait variation include *ra1* [4], *zagl1* [3], and *su1* [28]. These three genes are all ones for which the precise target trait under selection is unknown. Our associations provided candidate target traits for these genes that can be further investigated. There is one selected gene, *tga1*, for which our previous studies [13], [14] did not detect any significant associations with teosinte trait variation. This may not be all that surprising, given that we did not assay the target trait of *tga1*, the extent to which the kernel is encased by its stony casing [29]. Overall, these prior results suggest that selected genes, for which the evidence for selection is strong, often show associations with trait variation in teosinte.

While we have had some success in the present study at detecting associations between selection-candidates and teosinte trait variation, there are two classes of issues that if addressed could lead to further success. One possible issue is that the selection-candidates have little or no effect on trait variation in teosinte since the selection-candidates include a high number of false-positive or neutral genes because of insufficiencies in the selection screens. The other possibility is that these selection-candidates do in fact control domestication or improvement traits, but insufficiencies in the association analyses prevented us from detecting the associations. Below we explore these two explanations for our results.

There are many reasons why the association analyses could have limited success in detecting an association between a valid selection-candidate gene and trait variation in teosinte. First, the relevant trait may not have been assayed. Although the 32 traits that were assayed include many aspects of morphology that underwent dramatic change during domestication [26], other possible traits under selection such as kernel amino acid composition or kernel palatability were not assayed. Second, the traits controlled by the selection-candidates might have low heritabilities such that our sample sizes were too small to detect the association. Third, the SNPs assayed in the association analyses could be too distant from (or not in linkage disequilibrium with) the functional polymorphism in the gene. Fourth, the functional variants in the selection-candidate genes might exist at too low a frequency in teosinte populations to be detected by association mapping. It is possible that selection acted on rare de novo mutations, which would most likely be absent from the teosinte populations assayed in this analysis. Haplotype-based association mapping could possibly address these last two points, however the low number of SNPs per gene, the lack of phased data, and the low linkage disequilibrium within teosinte (A. L. Weber and J. F. Doebley, unpublished results) make this strategy difficult to implement and unlikely to yield more power. We believe that the issues listed above contribute to why the selection-candidates performed no better than random genes in the association analyses.

A second explanation for the small number of associations detected is that the selection-candidates include a high proportion of false positives or neutral genes. There are several reasons to suspect that this is the case for the selection-candidates assayed in this paper. Recently, Yamasaki *et al.* (2008) found that approximately 50% of the top 20 selection-candidate genes identified by Wright *et al.* (2005) are likely to be false positives [30]. This result is consistent with the average posterior probability of ∼53% that the gene experienced selection for the top 20 selection-candidate genes initially identified by Wright *et al.* (2005). Factors contributing to the high false positive rate include use of short amplicons (∼300 bp), use of a permissive *P*-value (0.1) for rejection of the null or neutral hypothesis [31], no correction for multiple tests, and an inadequate model of the domestication process in the coalescence simulation-based tests. Therefore, it is reasonable to assume that at least 50% of our selection-candidate genes did not undergo selection and have little or no effect on trait variation in teosinte.

This study was motivated by the concept that selection scans provide a useful entry point for the identification of genes that contribute to phenotypic changes during crop domestication and improvement [2]. While the concept remains valid, our results suggest that the practice may be challenging. Our ability to detect several significant associations suggests that association mapping in the ancestral population can be used as an initial step to infer the function of some selection-candidate genes. However in order for this approach to be more effective, the issues discussed above will have to be addressed.

## Materials and Methods

### Teosinte Sample

Two association mapping panels of Balsas teosinte (*Zea mays* ssp. *parviglumis)* plants were examined. Panel A includes plants sampled from 74 local teosinte populations found throughout Mexico [13]. In total, 592 plants were grown (8 plants per population). Panel A was grown in Hawaii on the island of Molokai, during the winter of 2002–2003. The field was divided into randomized plots, where each plot contained four individuals from the same population. Eight individuals with large amounts of missing data were dropped from analysis, resulting in a population of 584. Panel B includes plants sampled from 34 local populations found throughout the central Balsas river drainage in Mexico [14]. Thirty plants from each population were planted resulting in a total of 1020 plants. Panel B was grown in Tapachula, Nayarit, Mexico during the winter of 2004–2005. The plants were planted in a completely randomized design. Two hundred and three individuals with large amounts of missing data were dropped from analysis, resulting in a population of 817.

### Phenotypes and Genotypes

Seventeen phenotypes were measured on the plants in Panel A and 31 phenotypes were measured on the plants in Panel B (Table 1; Table S1, Supplementary section). These phenotypes included those that assayed flowering time, inflorescence architecture, plant architecture, vegetative morphology and kernel composition. SNPs from a set of selection-candidate genes identified through selection screens [5], [6] were developed using previously published sequence alignments for these genes (http://www.panzea.org). Discovery panels were variable due to the fact that the alignments came from diverse sources [5], [6]. The selection-candidate discovery panels contained a minimum of five, and on average ∼13 geographically diverse teosinte inbred individuals. The discovery panels for the random genes contained on average ∼11 geographically diverse teosinte inbred individuals however, there were several instances where no teosinte individuals were included in the discovery panel. Criteria for selection of the SNPs included (1) a minumum of 20 bp on one side of the target SNP that was devoid of polymorphism to allow for primer design, (2) base-call quality scores suggesting that the SNP of interest was not an artifact, and (3) a minor allele that was present in at least two individuals within the discovery panel [13]. In total, 56 SNPs in 26 selection-candidate genes were assayed in Panel A and 75 SNPs in 35 selection-candidate genes were assayed in Panel B (Table S2, Supplementary section). SNPs from randomly selected ESTs were used to measure population structure as previously described [13], [14].

### Association Mapping

A mixed linear model was used to test for SNP-trait associations: 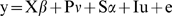, where y was a vector of phenotypic values, ν was a vector of fixed effects regarding population structure, α was the fixed effect for the SNP, u was a vector of the random effects pertaining to recent coancestry, and e was a vector of residuals. P was a matrix of principal component vectors, S was the vector of genotypes at the candidate marker, and I was an identity matrix. The Xβ term was only included in the model when testing associations in Panel A; β was a vector of fixed effects concerning the position of plants within the field and X included a row, a column, and a row-column interaction term. The variances of the random effects were assumed to be 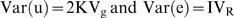, where K was the kinship matrix consisting of the proportion of shared allele values, I was an identity matrix, V_g_ the genetic variance and V_R_ the residual variance. The above model was used to test all possible SNP-trait pairs. Residuals indicated that no transformation of phenotypic values was necessary with the exception of percent paired spikelets (PASP) and percent yoked fruitcases (YKFC), both of which underwent square root transformations.

### E-Northern

We downloaded the February 6^th^ 2008 release of maize mRNA ESTs (dbEST) from the Plant GDB website (www.plantgdb.org). From the 234 EST libraries in the database, we selected a subset of 57 based on count number and tissue type. All normalized and subtracted EST libraries were dropped from analysis. We blasted a subset of our genes against the 514,104 maize ESTs from these chosen libraries. These libraries were categorized into four tissue types: ear, kernel, tassel, and vegetative tissue (Table S4, Supplementary section). Blast hits were filtered for an expected value (e-value) of less than or equal to 10^−30^, a blast alignment length of greater than or equal to 100 basepairs and a percent identity of greater than or equal to 91%. Expression level was then converted to parts per million based on the blast hit count and the number of ESTs queried for that particular tissue type.

### Permutation Tests

In order to compare the number of significant associations detected between teosinte trait variation and SNPs in the selection-candidate genes to that detected with SNPs in genes chosen at random from the genome, we used permutation tests. Molecular population genetics statistics were estimated using DnaSP [32] and compared between the two groups to verify that they were similar. For both Panels A and B, a subset of SNPs (56 in Panel A and 75 in Panel B) was selected from the random SNPs previously assayed in these panels [13], [14]. In order to select a group of SNPs comparable to those assayed for the selection-candidate genes, SNPs were selected based on major allele frequency and the number of SNPs assayed per gene. These SNPs were then tested for association with all traits measured. The *P-*values of these tests, as well as all tests for the selection-candidate genes were permuted within each of the two teosinte association mapping panels. Ten thousand permuted datasets were generated. For each permutated dataset, a chi-squared test was performed to ask if either the random gene or selection-candidate SNPs showed an excess of significant tests relative to each other. The chi-squared statistic on the unpermuted data was then compared with the distribution of chi-squares for the permuted datasets to determine the *P*-value of the permutation test. The permutation test was conducted once defining significant associations as those with *P*<0.05 and a second time defining significant associations as those with *P*<0.01.

## Supporting Information

Table S1 List of phenotypes without interesting associations.
(0.05 MB PDF)

Table S2 List of candidate genes assayed and corresponding references.
(0.10 MB PDF)

Table S3 A summary of the marker-trait associations.
(0.05 MB PDF)

Table S4 Expressed sequence tag (EST) libraries included in the e-northern analysis.
(0.04 MB PDF)
