# Supplementary material for: Using Association Mapping in Teosinte to Investigate the Function of Maize Selection-Candidate Genes
Source: PLoS One. 2009 Dec 9;4(12):e8227. doi: 10.1371/journal.pone.0008227 (PMC2785427; doi:10.1371/journal.pone.0008227)
Supplement: Table S1 — List of phenotypes without interesting associations. (0.05 MB PDF) [file pone.0008227.s001.pdf]

Table S1: List of phenotypes without interesting associations.

Group[a]	Trait	Description[b]	Units	Study
FWTM	LFNM (leaf number)	Number of leaves on the main stalk with the first leaf above ground being counted as leaf one	count	Panel B
FWTM	PLHT (plant height)	Length of the primary stalk from the ground to the tip of the primary tassel	cm	Panels A & B
FWTM	SILK (days to silk)	Days from planting to first visible silks on a single plant	days	Panels A & B
INFL	FCLN (fruitcase length)	Length of the female and hermaphroditic portions divided by the number of cupules in those portions	mm	Panel B
INFL	FELN (proportion of female ear length)	Length of the female and hermaphroditic portions of the basal-most ear on the lateral branch divided by the sum of the female, hermaphroditic and male lengths of the basal-most ear on the lateral branch	proportion	Panels A & B
INFL	FERL (female ear length)	Length of the female and hermaphroditic portions of the basal-most ear on the lateral branch	mm	Panels A & B
INFL	LIBN (lateral inflorescence branch number)	Number of branches in the tassel or inflorescence not including the central spike that terminates the lateral branch	count	Panels A & B
INFL	LILN (lateral inflorescence length)	Length of the peduncle and inflorescence terminating the lateral branch	cm	Panel B
INFL	NDFC (percent non-disarticulating fruitcases)	Percent cupulate fruitcases that did not fully disarticulate; this trait was measured on bulk seed harvested from the mature plant	percent	Panel B
INFL	PASP (percent paired spikelets)	Percent cupulate fruitcases that contain two pistillate spikelets; this trait was measured on bulk seed harvested from the mature plant	percent[c]	Panel B

Group[a]	Trait	Description[b]	Units	Study
INFL	PRFI (proportion of female internodes)[d]	Ratio of female cupules (including hermaphroditic cupulate fruitcases) in the basal-most ear on the lateral branch to the total number of internodes that produced identifiable male or female spikelets; no internodes present in the branches of the ear were included in analysis	percent	Panels A & B
INFL	PSIN (percent male internodes)	Fraction of the male internodes in the inflorescence that terminates the lateral branch	percent	Panel B
INFL	STAM (staminate score)	Fraction of the male spikelets in the inflorescence that terminates the lateral branch	percent	Panels A & B
INFL	TBN (tassel branch number)	Number of branches on the main tassel	count	Panel B
INFL	YKFC (percent yoked fruitcases)	Percent cupulate fruitcases that are yoked; this trait was measured on bulk seed harvested from the mature plant	percent[c]	Panel B
KERN	FRLYS (free lysine content)	Amount of free lysine per standardized weight of seeds	ppm	Panel A
KERN	PRCT (protein content)	Percent protein per gram of seed at zero moisture (dry matter)	percent	Panels A & B
PLNT	BRLN (branch length)	Length of the lateral branch not including the length of the inflorescence and peduncle at the tip of that branch	cm	Panels A & B
PLNT	LBIL (mean lateral branch internode length)	Length of the lateral branch divided by the number of internodes in that branch	cm	Panels A & B
PLNT	LBIN (lateral branch internode number)	Number of internodes that compose the lateral branch	count	Panel B
PLNT	PROL (prolificacy)	Number of ears along the lateral branch	count	Panel B
PLNT	TILL (tiller number)	Number of tillers at time of pollen shed	count	Panels A & B

Group[a]	Trait	Description[b]	Units	Study
VEGT	BLLN (blade length)	Length of the leaf blade for the basal-most leaf on the lateral branch	cm	Panel B
VEGT	CULM (culm diameter)	Maximum diameter of the main culm measured at the midpoint of the internode between the third and fourth node above ground	mm	Panel B
VEGT	LFWH (leaf width)	Maximum width for the longest leaf on the main stalk	cm	Panel B
VEGT	SHLN (sheath length)	Length of the sheath for the basal-most leaf on the lateral branch	cm	Panel B

[a]The trait groups are designated as: inflorescence architecture (INFL), plant architecture (PLNT), flowering time (FWTM), vegetative morphology (VEGT) and kernel composition (KERN) traits.
[b]All lateral branch traits were measured on the upper-most well-formed lateral branch in Panel A and the second lateral branch from the top of the plant in Panel B.
[c]A square root transformation was performed on the trait values.
[d]Proportion of female internodes (PRFI) is equivalent to proportion of female cupules (PRFCP) in Weber *et al.* 2007.
