# Supplementary material for: Using Association Mapping in Teosinte to Investigate the Function of Maize Selection-Candidate Genes
Source: PLoS One. 2009 Dec 9;4(12):e8227. doi: 10.1371/journal.pone.0008227 (PMC2785427; doi:10.1371/journal.pone.0008227)
Supplement: Table S2 — List of candidate genes assayed and corresponding references. (0.10 MB PDF) [file pone.0008227.s002.pdf]

Table S2: List of candidate genes assayed.

Gene[a]	Description	Source	Number of SNPs in Panel A	B
AY103840	Homologous by sequence to *KAKTUS* which regulates the number of endoreduplication cycles in Arabidopsis (El Refy *et al.* 2003)	Wright *et al.* 2005	2	3
AY104037	Homologous by sequence to a cytosolic aconitase that participates in the glyoxylate cycle in pumpkin (Hayashi *et al.* 1995)	Wright *et al.* 2005	2	2
AY104065 (*ZmDWF1*)	A gene involved in cell elongation in maize (Tao *et al.* 2004)	Wright *et al.* 2005	0	1
AY104090	Unknown	Wright *et al.* 2005	1	1
AY104439	An indol-3-glycerol phosphate lyase involved in metabolism important for pathogen defense in maize (Kramer and Koziel 1995).	Wright *et al.* 2005	0	1
AY104530	Homologous to *SNFL1*, a serine/threonine kinase in Sorghum (Annen and Stochaus 1998).	Wright *et al.* 2005	1	2
AY104948	Homologous by sequence to *ARF2* a pleiotropic developmental regulator in Arabidopsis (Ulmasov *et al.* 1999)	Wright *et al.* 2005	1	1
AY105060	Weakly similar a Myb-like DNA-binding domain SHAQKYF class family protein in Tetrahymena thermophila (Eisen *et al.* 2006)	Yamasaki *et al.* 2005	4	3
AY105809	Homologous by sequence to a putative taxadienol acetyl transferase-like protein in rice (Sasaki *et al.* 2002)	Wright *et al.* 2005	0	1
AY105958	Homologous by sequence to a putative auxin-induced protein in rice (Saski *et al.* 2002)	Wright *et al.* 2005	0	1

Gene[a]	Description	Source	Number of SNPs in Panel A	B
AY106111 (*HXK1*)	A hexokinase that phosphorylates glucose and fructose (Galina *et al.* 1995)	Wright *et al.* 2005	1	1
AY106371	Putative methyl binding domain protein (Chandler *et al.* 2001)	Yamasaki *et al.* 2005	5	5
AY106496	Unknown	Wright *et al.* 2005	0	1
AY106600	Homologous by sequence to a putative adenylosuccinate synthetase in rice (Buell *et al.* 2005)	Wright *et al.* 2005	1	2
AY106616	Homologous by sequence to a predicted ankyrin repeat-like protein (International rice genome sequencing project 2005)	Yamasaki *et al.* 2005	6	7
AY106970	Unknown	Wright *et al.* 2005	0	1
AY107195	Homologous by sequence to *ARF1,* a transcription factor involved in auxin regulation in Arabidopsis (Ulmasov *et al.* 1997)	Yamasaki *et al.* 2005	7	6
AY107228	Dihydrodipicolinate synthase (*DHPS*) an enzyme involved in amino acid biosynthesis in maize (Frisch *et al.* 1991)	Wright *et al.* 2005	1	3
AY107475	Similar in sequence to chitinase (*chI*) a protein involved in plant defense in maize (Tiffin 2004)	Wright *et al.* 2005	0	1
AY107903	Homologous by sequence to a putative U4/U6.U5 tri-snRNP-associated 65 kDa protein in rice (International Rice Genome Sequencing Project 2005)	Wright *et al.* 2005	1	1
AY107907	Homologous by sequence to a putative chorismate mutase in rice (International Rice Genome Sequencing Project 2005)	Wright *et al.* 2005	0	1
AY107952	Homologous by sequence to a putative fruit protein in kiwifruit (Ledger and Gardner 1994)	Yamasaki *et al.* 2005	7	7

Gene[a]	Description	Source	Number of SNPs in Panel A	B
AY108178	Homologous by sequence to *ZEITLUPE* a circadian clock gene in Arabidopsis (Somers *et al.* 2000)	Yamasaki *et al.* 2005	1	2
AY108187	Unknown	Wright *et al.* 2005	0	1
AY108246	Unknown	Wright *et al.* 2005	1	1
AY108481	Homologous by sequence to a methyltransferase-like protein in rice (International Rice Genome Sequencing Project 2005)	Wright *et al.* 2005	1	2
AY108543	Homologous by sequence to a putative early responsive to dehydration stress protein (International Rice Genome Sequencing Project 2005)	Wright *et al.* 2005	1	1
AY108876	Homologous by sequence to a putative amino acid transporter in rice (International Rice Genome Sequencing Project 2005)	Yamasaki *et al.* 2005	2	3
AY110082	Homologous by sequence to a putative heat shock protein in rice (Feng *et al.* 2002)	Wright *et al.* 2005	2	3
AY110109	Unknown	Yamasaki *et al.* 2005	2	1
AY111438	Unknown	Wright *et al.* 2005	2	2
AY111546	Unknown	Wright *et al.* 2005	1	3
MAGI_107879	A MYB protein (Martin and Paz-Ares 1997)	Michael D. McMullen, personal communication	1	1
MAGI_69014	A MYB protein (Martin and Paz-Ares 1997)	Michael D. McMullen, personal communication	1	2

Gene[a]	Description	Source	Number of SNPs in Panel A	B
MAGI_84234	A MYB protein (Martin and Paz-Ares 1997)	Michael D. McMullen, personal communication	1	1

[a]The publicly available sequence from which the amplicon primers were designed off of is listed.

References

ANNEN, F., and J. STOCKHAUS 1998 Characterization of a *Sorghum bicolor* gene family encoding putative protein kinases with a high similarity to the yeast SNF1 protein kinase. Plant Mol. Biol. **36:** 529-539.

BUELL, C. R., Q. YUAN, S. OUYANG, J. LIU, W. ZHU, 2005 Sequence, annotation, and analysis of synteny between rice chromosome 3 and diverged grass species. Genome Res. **15:** 1284-1291.

CHANDLER, V. L., K. C. CONE, H. F. KAEPPLER and S. M. KAEPPLER, 2001 Sequences from the Plant Chromatin Consortium (NSF Plant Genome project 9975930).

EISEN, J. A., R. S. COYNE, M. WU, D. WU, M. THIAGARAJAN *et al.*, 2006 Macronuclear genome sequence of the ciliate *Tetrahymena thermophila*, a model Eukaryote. PLoS Biol. **4:** e286.

EL REFY, A., D. PERAZZA, L. ZEKRAOUI, J-G. VALAY, N. BECHTOLD *et al.*, 2003 The *Arabidopsis KAKTUS* gene encodes a HECT protein and controls the number of endoreduplication cycles. Mol. Gen. Genomics **270:** 403-414.

FENG, Q., Y. ZHANG, P. HAO, S. WANG, G. FU *et al.*, 2002 Sequence and analysis of rice chromosome 4. Nature **420:** 316-320.

FRISCH, D. A., A. M. TOMMEY, B. G. GENGENBACH, and D. A. SOMERS, 1991 Direct genetic selection of a maize cDNA for dihydrodipicolinate synthase in an Escherichia coli dapA-auxotroph. Mol. Gen. Genet. **228:** 287-293.

GALINA, A., M. REIS, M. C. ALBUQUERQUE, A. G. PUYOU, M. T. PUYOU *et al.*, 1995 Different properties of the mitochondrial and cytosolic hexokinases in maize roots. Biochem J. **309:** 105-112.

HAYASHI, M. L. DE BELLIS, A. ALPI and M. NISHIMURA, 1995 Cytosolic Aconitase Participates in the Glyoxylate Cycle in Etiolated Pumpkin Cotyledons. Plant Cell Physiol. **36:** 669-680.

INTERNATIONAL RICE GENOME SEQUENCING PROJECT, 2005 The map-based sequence of the rice genome. Nature **436:** 793-800.

KRAMER, V. C. and M. G. KOZIEL, 1995 Structure of a maize tryptophan synthase alpha subunit gene with pith enhanced expression. Plant Mol. Biol. **27:** 1183-1188.

LEDGER, S. E. and R. C. GARDNER, 1994 Cloning and characterization of five cDNAs for genes differentially expressed during fruit development of kiwifruit (*Actinidia deliciosa* var. *deliciosa*). Plant Mol. Bio. **25:** 877-886.

MARTIN, C. and J. PAZ-ARES, 1997 MYB transcription factors in plants. Trends Genet. **13:** 67-73.

SASAKI, T., T. MATSUMOTO, K. YAMAMOTO, K. SAKATA, T. BABA *et al.*, 2002 The genome sequence and structure of rice chromosome 1. Nature **420:** 312-316.

SOMERS, D. E., T. F. SCHULTZ, M. MILNAMOW and S. A. KAY, 2000 *ZEITLUPE* encodes a novel clock-associated PAS protein from *Arabidopsis.* Cell **101:** 319-329.

TAO, Y., J. ZHENG, Z. XU, X. ZHANG, K. ZHANG, *et al.*, 2004 Functional analysis of *ZmDWF1*, a maize homolog of the *Arabidopsis* brassinosteroids biosynthetic *DWF1/DIM* gene. Plant Sci. **167:** 743-751.

TIFFIN, P. 2004 Comparative evolutionary histories of chitinase genes in the genus Zea and Family Poaceae. Genetics **167:** 1331-1340.

ULMASOV, T., G. HAGAN and T. J. GUILFOYLE, 1997 ARF1, a transcription factor that binds to auxin response elements. Science **276:** 1865-1868.

ULMASOV, T., G. HAGAN and T. J. GUILFOYLE, 1999 Dimerization and DNA binding of auxin response factors. Plant J. **19:** 309-319.
