# Supplementary material for: Using Association Mapping in Teosinte to Investigate the Function of Maize Selection-Candidate Genes
Source: PLoS One. 2009 Dec 9;4(12):e8227. doi: 10.1371/journal.pone.0008227 (PMC2785427; doi:10.1371/journal.pone.0008227)
Supplement: Table S3 — A summary of the marker-trait associations. (0.05 MB PDF) [file pone.0008227.s003.pdf]

Table S3: A summary of the marker-trait associations.

Trait	Panel	$P < 0.05$	$P < 0.01$	Bonferroni Correction
BLLN	B	5	0	0
BRLN	A	2	0	0
BRLN	B	2	1	0
CULM	B	6	1	0
DSCT	A	2	0	0
DSCT	B	4	0	0
FCLN	B	3	3	0
FCWT	A	10	1	0
FCWT	B	4	0	0
FELN	A	1	0	0
FELN	B	0	0	0
FERL	A	4	2	0
FERL	B	5	0	0
FRLYS	A	5	0	0
LBIL	A	2	1	0
LBIL	B	3	1	0
LBIN	B	4	2	0
LFNM	B	0	0	0
LFWH	B	3	2	0
LIBN	A	2	1	0
LIBN	B	3	1	0
LILN	B	1	0	0
NDFC	B	5	1	0
NMFC	A	3	0	0
NMFC	B	5	0	0
OLCT	A	4	0	0
OLCT	B	5	0	0
PASP	B	3	2	0
PESP	B	4	1	1
PLHT	A	2	0	0
PLHT	B	4	1	0
POLL	A	4	0	0
POLL	B	6	3	1
PRCT	A	2	0	0
PRCT	B	1	0	0
PRFI	A	2	0	0
PRFI	B	1	0	0
PROL	B	1	0	0
PSIN	B	2	1	0
SHLN	B	3	1	0
SILK	A	3	0	0
SILK	B	5	3	0
STAM	A	2	1	0
STAM	B	0	0	0

Trait	Panel	$P < 0.05$	$P < 0.01$	Bonferroni Correction
TBN	B	2	1	0
TILL	A	1	0	0
TILL	B	6	3	0
YKFC	B	5	1	0
