# Supplementary material for: Using Association Mapping in Teosinte to Investigate the Function of Maize Selection-Candidate Genes
Source: PLoS One. 2009 Dec 9;4(12):e8227. doi: 10.1371/journal.pone.0008227 (PMC2785427; doi:10.1371/journal.pone.0008227)
Supplement: Table S4 — Expressed sequence tag (EST) libraries included in the e-northern analysis. (0.04 MB PDF) [file pone.0008227.s004.pdf]

Table S4: Expressed sequence tag (EST) libraries included in the e-northern analysis.

Library	Group	Count
1091 - Immature ear with common ESTs screened by Schmidt lab	Ear	9277
3524 - Mature pollen from Sheila McCormick's lab	Tassel	3942
3529 - 2 mm ear tissue from Schmidt and Hake labs	Ear	19258
486 - leaf primordia cDNA library from Hake lab	Veg	5867
487 - apical meristem cDNA library from Hake lab	Veg	684
496 - stressed shoot cDNA library from Wang/Bohnert lab	Veg	1306
603 - stressed root cDNA library from Wang/Bohnert lab	Veg	2023
605 - Endosperm cDNA library from Schmidt lab	Kernel	6565
606 - Ear tissue cDNA library from Schmidt lab	Ear	5516
614 - root cDNA library from Walbot Lab	Veg	10611
618 – Inbred Tassel cDNA Library	Tassel	3407
660 – Mixed stages of anther and pollen	Tassel	6444
683 - 14 day immature embryo from Hake lab (HS)	Kernel	1138
687 - Early embryo from Delaware	Kernel	4763
946 - tassel primordium prepared by Schmidt lab	Tassel	21232
947 - 2 week shoot from Barkan lab	Veg	8878
949 - Juvenile leaf and shoot cDNA from Steve Moose	Veg	10708
950 - Mature pollen from Sheila McCormick's lab	Tassel	427
953 - Immature ear with common ESTs screened by Schmidt lab	Ear	248
Corn W64A De*-B30 endosperm cDNA library	Kernel	75
CSU	Veg	13
E7PCR	Kernel	2496
Etiolated seedling	Veg	166
ISUM7	Veg	815
LCM-dissected maize shoot apical meristem cDNA	Veg	260736
Maize ear glume library	Ear	305
Maize Endosperm cDNA Library	Kernel	30553
Maize Glume cDNAs Library	Ear	2147
Maize inflorescence immature ear library	Ear	509
Maize ovary – Eveland 2007	Kernel	14822
membrane-free polysomes from endosperm	Kernel	632
QBA	Kernel	2449
QBB	Kernel	2578
QBC	Kernel	690
QBD	Ear	696
QBE	Veg	602
QBG	Kernel	2320
QBH	Veg	3098
QBI	Ear	3071
QBJ	Tassel	3206
QBK	Kernel	2170
QBL	Veg	2047
QBN	Ear	2548
QBO	Veg	593

Library	Group	Count
QBQ	Kernel	1720
QBS	Veg	1234
Salt stressed Zea mays leaves cDNA library	Veg	56
Salt stressed Zea mays roots cDNA library	Veg	652
UGA-ZmSAM-XZ1	Veg	64
UGA-ZmSAM-XZ2	Veg	30972
Vegetative meristem	Veg	134
Zea mays early female inflorescence	Ear	14
Zea mays embryo sac cDNA library	Kernel	10738
Zea mays mesophyll cell	Veg	10
Zea mays sperm cell cDNA library	Tassel	5174
Zm03_AAFC_ECORC_cold_stressed_maize_seedlings	Veg	951
Zm04_AAFC_ECORC_cold_stressed_maize_seedlings	Veg	754
